# Radiology departments as COVID-19 entry-door might improve healthcare efficacy and efficiency, and emergency department safety

**DOI:** 10.1186/s13244-020-00954-8

**Published:** 2021-01-04

**Authors:** José M. García Santos, Juana M. Plasencia Martínez, Pablo Fabuel Ortega, Marina Lozano Ros, María Carmen Sánchez Ayala, Gloria Pérez Hernández, Pedro Menchón Martínez

**Affiliations:** 1 Radiology Department, University General Hospital Morales Meseguer, C/ Marqués de Los Vélez, s/n., 30008 Murcia, Spain; 2 Primary Care Health Center Vistabella-La Flota, 6th Health Area, Comunidad Autónoma de la Región de Murcia, Murcia, Spain; 3 Primary Care Health Center Molina-Jesús Marín, 6th Health Area, Comunidad Autónoma de la Región de Murcia, Murcia, Spain; 4 Neumology Section, University General Hospital Morales Meseguer, Murcia, Spain; 5 Medical Direction, 6th Health Area, Comunidad Autónoma de La Región de Murcia, University General Hospital Morales Meseguer, Murcia, Spain

**Keywords:** Coronavirus, COVID-19, Radiology, Emergency medicine, Primary health care

## Abstract

**Background:**

Possible COVID-19 pneumonia patients (ppCOVID-19) generally overwhelmed emergency departments (EDs) during the first COVID-19 wave. Home-confinement and primary-care phone follow-up was the first-level regional policy for preventing EDs to collapse. But when X-rays were needed, the traditional outpatient workflow at the radiology department was inefficient and potential interpersonal infections were of concern. We aimed to assess the efficiency of a primary-care high-resolution radiology service (pcHRRS) for ppCOVID-19 in terms of time at hospital and decision’s reliability.

**Methods:**

We assessed 849 consecutive ppCOVID-19 patients, 418 through the pcHRRS (home-confined ppCOVID-19 with negative—group 1- and positive—group 2-X-rays) and 431 arriving with respiratory symptoms to the ED by themselves (group 3). The pcHRRS provided X-rays and oximetry in an only-one-patient agenda. Radiologists made next-step decisions (group 1: pneumonia negative, home-confinement follow-up; group 2: pneumonia positive, ED assessment) according to X-ray results. We used ANOVA and Bonferroni correction, Student T, Chi^2^ tests to analyse changes in the ED workload, time-to-decision differences between groups, potential delays in patients acceding through the ED, and pcHRRS performance for deciding admission.

**Results:**

The pcHRRS halved ED respiratory patients (49.2%), allowed faster decisions (group 1 vs. home-discharged group 2 and group 3 patients: 0:41 ± 1:05 h; 3:36 ± 2:58 h; 3:50 ± 3:16 h; group 1 vs. all group 2 and group 3 patients: 0:41 ± 1:05 h; 5.25 ± 3.08; 5:36 ± 4:36 h; group 2 vs. group 3 admitted patients: 5:27 ± 3:08 h vs. 7:42 ± 5:02 h; all *p* < 0.001) and prompted admission (84/93, 90.3%) while maintaining time response for ED patients.

**Conclusions:**

Our pcHRRS may be a more efficient entry-door for ppCOVID-19 by decreasing ED patients and making expedited decisions while guaranteeing social distance.

## Key points

- The pcHRRS provided safe and efficient support for home-confined possible COVID-19 pneumonia.
- The pcHRRS was easy to set up and succeeded very soon.
- A radiology triage reduced both workload and time in the emergency department.
- The pcHRRS potentially decreased infection hazards for patients and health professionals.

## Background

In the Region of Murcia, Spain, general practitioners (GPs) see patients within a primary care network constituted by nine health areas with their own referral hospital. Each area includes basic health areas pivoting on primary care health centres. Given the previous experiences in Italy and other Spanish regions, with the arrival of the COVID-19 wave in February 2020, regional health authorities put forward a strategy based on primary care health centres GPs keeping patients with mild clinical presentation at home, similarly to the European recommendations [1]; at the Region of Murcia, they controlled more than 26,800 symptomatic cases suspected or diagnosed with COVID-19 and more than 34,600 asymptomatic contacts at home, only by close telephone follow-up [2]. But soon multiple conditioning factors came up and urged us to set up a secure primary care high-resolution radiology service (pcHRRS) in our health area, out of the emergency department (ED). These factors were the recommendations for chest X-rays even in the more resource-constrained scenario [3], the high volume of home-confined patients, the prevalence of dyspnoea in many patients, and the growing number of subjects asked by phone to go to the emergency or the radiology departments (RD) at the referral hospital without previous warning. The pcHRRS operated with a specific RD team 12-h daily, being at the same time the ED entry-door when appropriated. It was designed to offer GPs prompt objective respiratory clinical information from their home-based patients; to immediately transfer patients with pneumonia to the ED; to avoid overwhelming arrivals of respiratory patients to the ED; and, finally, to pilot and export the idea to the other health areas. Assuming the potential infection risk at overcrowded environments [4], we hypothesised that the pcHRRS would provide a much more efficient safer care. For that purpose, we aimed to analyse the pcHRRS efficiency in terms of reduced ED workload, waiting times, and admission triage through a simple radiology algorithm.

## Methods

This cross-sectional study followed the SQUIRE guidelines and was approved by our hospital ethical committee (C.I. EST: 55/20). Patients’ informed consent was waived.

### Background

The pcHRRS started on 26 March 2020 and continues active since then. The patient recruitment started on March 26th and ended on 17 April 2020. Our health area coped with the highest COVID-19 burden (26.4%) at the Region of Murcia. There were 233 confirmed SARS-CoV-2 infections, 4004 accumulated possible cases and 4687 contacts by 17 April 2020. In the same period, 152 COVID-19 patients were hospitalised, which was also the highest number in all health areas (23.4%).

Considering the usual number of patients seen in our ED before the pandemic start (e.g. 20–26 February: 1657 patients) and a ratio of respiratory/non-respiratory patients close to 1 (0.94, 206/218, 20–26 March) during the first epidemic week, we expected 118 [(1657/2)/7] or more daily respiratory emergencies during the epidemic wave. The average waiting time for patients with suspected respiratory infection at the ED during the epidemic wave was 5:48 h. Despite the ED having established separated ways for respiratory and non-respiratory patients, COVID-19 infection risk would presumably increase if health care was provided through the usual indoors overcrowded ED environment with extended waiting times [4]. Accordingly, we designed a straightforward specific radiology algorithm trying to keep the vast majority of possible pneumonia (ppCOVID-19) patients out of the ED, through an individualised short-time service while being highly effective for triaging need for admission.

### Intervention

#### pcHRRS characteristics

The pcHRRS provided chest X-rays and oximetry, making unnecessary a direct GP-to-patient contact. To be useful, the pcHRRS had to be (1) relevant: by deciding next steps; (2) accessible: available in less than 24 h for any home-confined patient; (3) swift: less than 15-min RD workflow without waiting time on an only-one-patient appointment and expedited electronic report for the GP or the ED; and (4) safe: by reducing risks of (a) staff infections: radiographers and nurses in charge of the oximetry and patient’s navigation avoided close contacts wearing the available personal protective equipment; (b) patients infections: they knew in advance how to reach the radiology room limiting interactions with other patients; barrier and hygiene resources were always available; and (c) wrong communication with the ED: COVID-19 ED physicians were fully aware of patient’s management through the pcHRRS.

#### pcHRRS resources (Fig. 1, Additional file 1: Fig. 1)

**Fig. 1:**
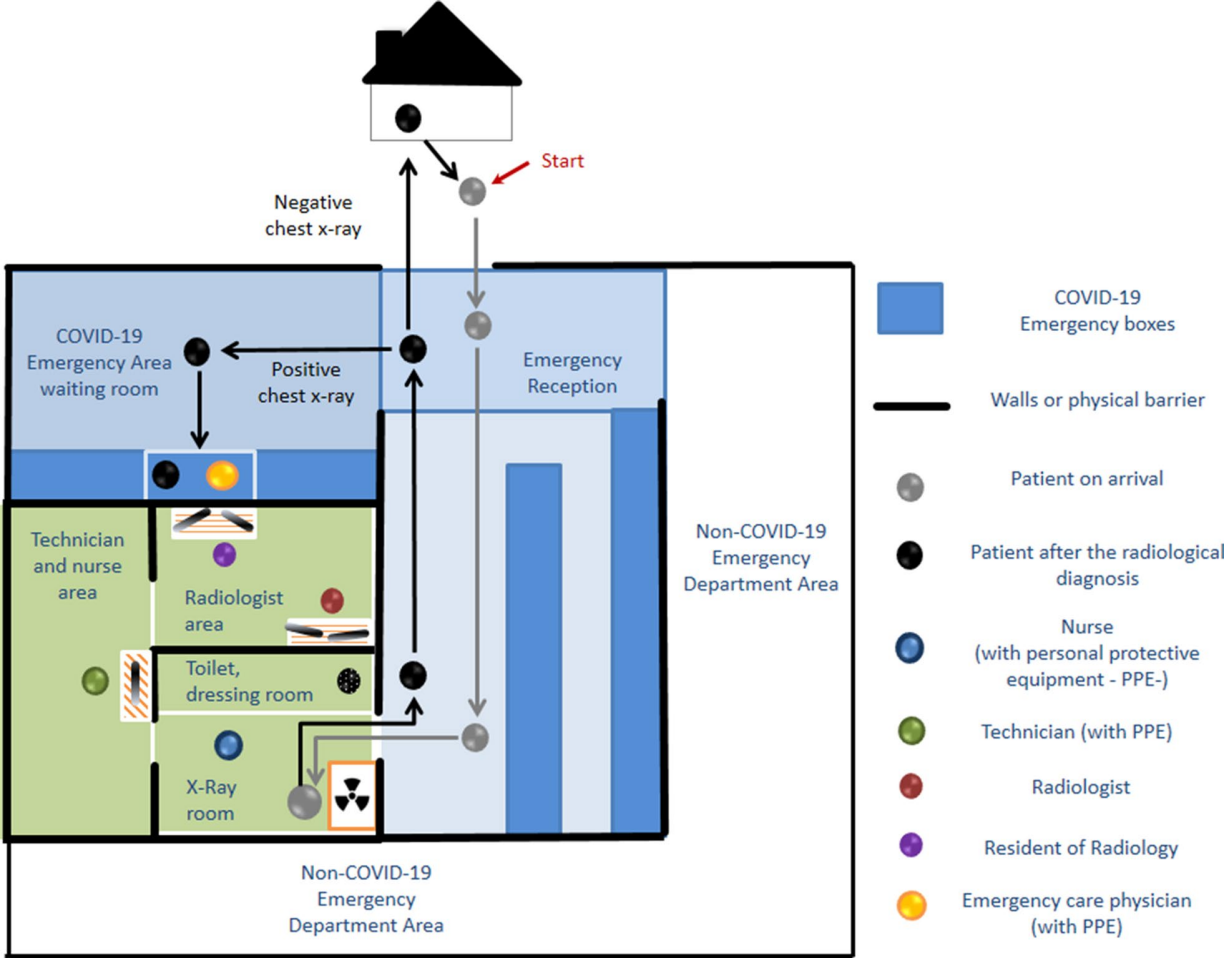
Primary care high-resolution radiology service (pcHRRS) workflow. The pcHRRS was integrated in the emergency radiology area (green background) and in the COVID-19 area at the emergency department (blue background). PPE: Personal protective equipment; RDR: Radiology Department Radiographer

1. General practitioners**.** Suspected or confirmed COVID-19 cases were interviewed by telephone every day. GPs had to rule out ppCOVID-19 if fever remained more than 6–7 days or persistent respiratory symptoms or worsening of respiratory or general condition at any time (especially dyspnoea). Those patients were appointed to the pcHRRS.
2. Specific electronic agenda. GPs could schedule chest X-rays into the radiological information system from 9:00 a.m. to 9:00 p.m., every 15 min Monday to Sunday, and every 30 min on weekends and holidays.
3. COVID-19 radiology room. Short street access room with a robotised X-ray digital 3D tomosynthesis (3DDT) and immediate PACS archiving, limiting any patient-to-patient and patient-to-staff interactions.
4. Radiology department workflow
  - Administrative staff. As soon a patient was appointed, a radiology secretary phoned encouraging him to attend the appointment and giving instructions for a safe access to the radiology room (Additional file 1: Fig. 2). A radiology resident played that role on weekends and holidays.
  - Reception. Upon arrival, the patient warned the reception staff that he was coming through the pcHRRS and was provided with a surgical mask. Relatives generally waited in the street to avoid person-to-person interactions. The reception staff checked that patients knew how to reach the radiology room, preventing random navigation through other areas.
  - Radiology department radiographers and nurses. When arriving to the radiology room, the patient proceeded immediately when the door was open. They were instructed to clean their hands with hydro-alcoholic solution, alcohol or to put gloves on, depending on daily resources. Within the room they received remote instructions from the radiographer so as to do the posteroanterior and lateral chest X-ray views, or a lateral view and 3DDT. Then, the nurse performed oximetry and informed the radiology resident. Once the radiologists assessed the X-rays and decided the next step, the nurse informed the patient and, when needed, went with him to the ED admission point, preventing him from accidentally leaving the pcHRRS or random navigations, and avoiding delays and X-ray repetitions. When occasional delays made an arriving patient to wait outside the room, the patient within stayed in the changing room while the radiographer cleaned every contacted element. Once the decision was made, the technical staff cleaned the changing room and started again.
  - Radiologists. A resident and a staff radiologist worked close to the COVID-19 room, allowing a fast and direct communication always maintaining a safe distance with radiographers and nurses, and between themselves. The radiology resident, who was the only additional pcHRRS personnel resource, (1) assessed chest X-rays and was allowed to send the patient to the ED when sure about signs of pneumonia; abnormal X-ray with findings different from pneumonia where handled as usual (Additional file 1: Fig. 3); (2) drafted structured reports to be eventually validated by the radiologist; he used standardised radiology information according to scientific recommendations [5], also including oximetry results and the patient’s final destination (Additional file 1: Fig. 4); (3) phoned the ED COVID-19 physicians warning about abnormal X-rays; (4) recorded and followed up every case; (5) recorded every pcHRRS incident; and (6) played the administrative role on weekends and holidays, being the reason for the 30-min time slots on those days. The consultant radiologist supervising the pcHRRS on weekdays was one of our regular on-duty emergency radiologists in charge and the on-call radiologist in charge on weekends and holidays. They guaranteed a correct workflow, supervised the radiology resident and validated radiology reports in non-conclusive and normal cases, and whenever requested by the resident. Patients were always referred to the ED when chest X-rays were regarded as abnormal.

Including the pcHRRS intermixed with our emergency workflow could have delayed our usual management of ED patients, so the on-call radiology team and all radiographers shift were systematically encouraged to keep all their attention on both patients workflows.

5. Emergency department. A COVID-19 physician evaluated every pcHRRS patient with radiological findings of pneumonia. The workflow was streamlined since the patient didn’t need a triage and had reported X-rays and oximetry.
6. Crisis committee. The Head of the RD, the Primary Care Network Director and the Medical Director of our health area, and one of the emergency radiologists met every day to know the number of involved patients, clinical results and incidents, so as to make changes on the fly. When required, the RD Supervisor and the Administrative Coordinator, and the ED COVID-19 Medical Coordinator attended the initial meetings.

For our purposes, all consecutive pcHRRS and the ED patients with respiratory infection symptoms were retrospectively studied from 5 days before the pcHRRS started. All pcHRRS patients underwent conventional chest X-rays with posteroanterior and lateral views. A systematic assessment by 3DDT and oximetry was implemented later in the pcHRRS.

### Statistical analysis

Patients were stratified in: group 1 (G1: pcHRRS; normal X-rays; returning home); group 2 (G2: pcHRRS; X-ray pneumonia findings; referred to the ED); and group 3 (G3: ED; respiratory infection symptoms according to the ED physician). For G1, the process length was the period between the pcHRRS appointment time and the radiology report validation time; for G2 and G3, it went from the arrival time to the ED to the clinical report signature time. Any G1 patient deciding to seek medical advice at the ED after leaving the pcHRRS was included in G3. Patients leaving or requesting voluntary medical discharge were included in the number of patients attended, but excluded from the time analysis, as this variable was lacking. Patients’ inflow was represented by daily absolute and relative frequencies, and the total accumulated frequency for all groups, and the daily ratio of hospitalised patients for groups 2 and 3. To assess the potential impact of the pcHRRS on the ED workflow, we mined the electronic X-ray request time, X-ray examination time and radiology report validation time in every ED patient during the five days before (173 patients) and after (107 patients) the pcHRRS was launched. Finally, when we began to write this manuscript, we had all radiological and laboratory data records for the first 212 consecutive pcHRRS patients. SARS-CoV-2 infection was confirmed by reverse transcription polymerase chain reaction (PT-PCR), serology, or both in 39/212 (17.45%) and ruled out in 86/212 patients. In 87/212 patients, the infection was ruled out, though results are now under review. For these patients, we assessed the proportions of normal, questionable and abnormal X-ray examinations, differences in age and gender among G1 and G2 patients as well as the radiology follow-up results depending on the initial X-ray results. Differences in oximetry measurements depending on the X-ray results were also analysed.

The ANOVA and Bonferroni correction, Student T and Chi^2^ tests were applied when appropriate. Statistically significant differences were assumed when *p* < 0.05. The analysis was performed with the IBM Statistics SPSS 20 software. For graphs, we also used the Excel Microsoft Office 365 software.

## Results

A top of 1494 confirmed and 5010 possible cases/week was reached in 23–29 March 2020, with a maximum of 119 confirmed cases/day on 25 March 2020. We considered that day to be the peak of the epidemic wave. The pcHRRS started on 26 March 2020 and has been active since then. From March 26th to 17 April 2020, 418 and 431 respiratory infection patients were seen through the pcHRRS and the ED, respectively. Ten scheduled patients did not attend the pcHRRS appointment. Those 418 patients accounted for 9.86% of the active confirmed or possible accumulated cases (233 and 4004 patients, respectively) followed up by telephone in that period of time, and 0.16% of our health area population (265.842 people).

The distribution of pcHRRS patients was: G1 325/418 (77.75%) and G2 93/418 (22.24%) patients (Additional file 1: Fig. 5). One patient with known fibrotic pulmonary lesions and other with a calcified granuloma where sent back home and included in G1. Among ED patients (G3), 224/431 (52%), 203/431 (47.10%) and 4/431 (0.93%) returned home, were admitted or refused admission, respectively. Eight pcHRRS patients encouraged to go home asked for ED assessment before being finally discharged. All of them went out from G1 (*n* = 317; 325 − 8) to be included in G3 (*n* = 439; 431 + 8) for the efficiency analysis; one G2 patient and four G3 patients refused medical attention at the ED and were excluded. Therefore, the final sample for our analysis was made up of 317 G1, 92 G2 and 435 G3 patients (Additional file 1: Fig. 5).

**Fig. 2:**
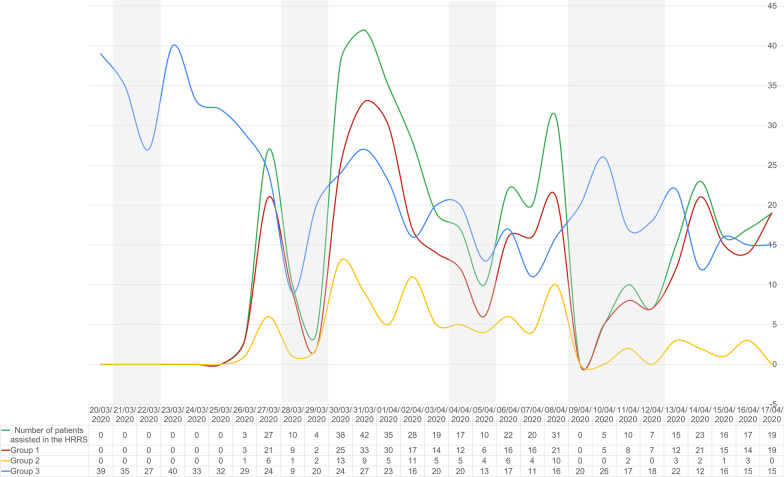
Daily respiratory infection patients flow during the epidemic wave in the Region of Murcia. Although patient recruitment began when the pcHRRS started on March 26, we show the number of patients seen in the emergency department (group 3) since March 20, so that the impact of the pcHRRS by decreasing the number of group 3 patients can be appreciated. (a) All primary care high-resolution radiology service (pcHRRS) patients (green line); (b) pcHRRS discharged patients (group 1, red line); (c) pcHRRS patients referred to the emergency department (ED) (group 2, yellow line); (d) patients arriving to the ED by themselves (group 3, blue line). Grey bands correspond to weekends and holidays. Patients seen within the pcHRRS (green line) exceeded the number of patients arriving to the ED (blue line) in the study window, except for holidays, when, despite being operational, patients were less referred to pcHRRS. Most patients seen at the pcHRRS went back home (red line)

**Fig. 3:**
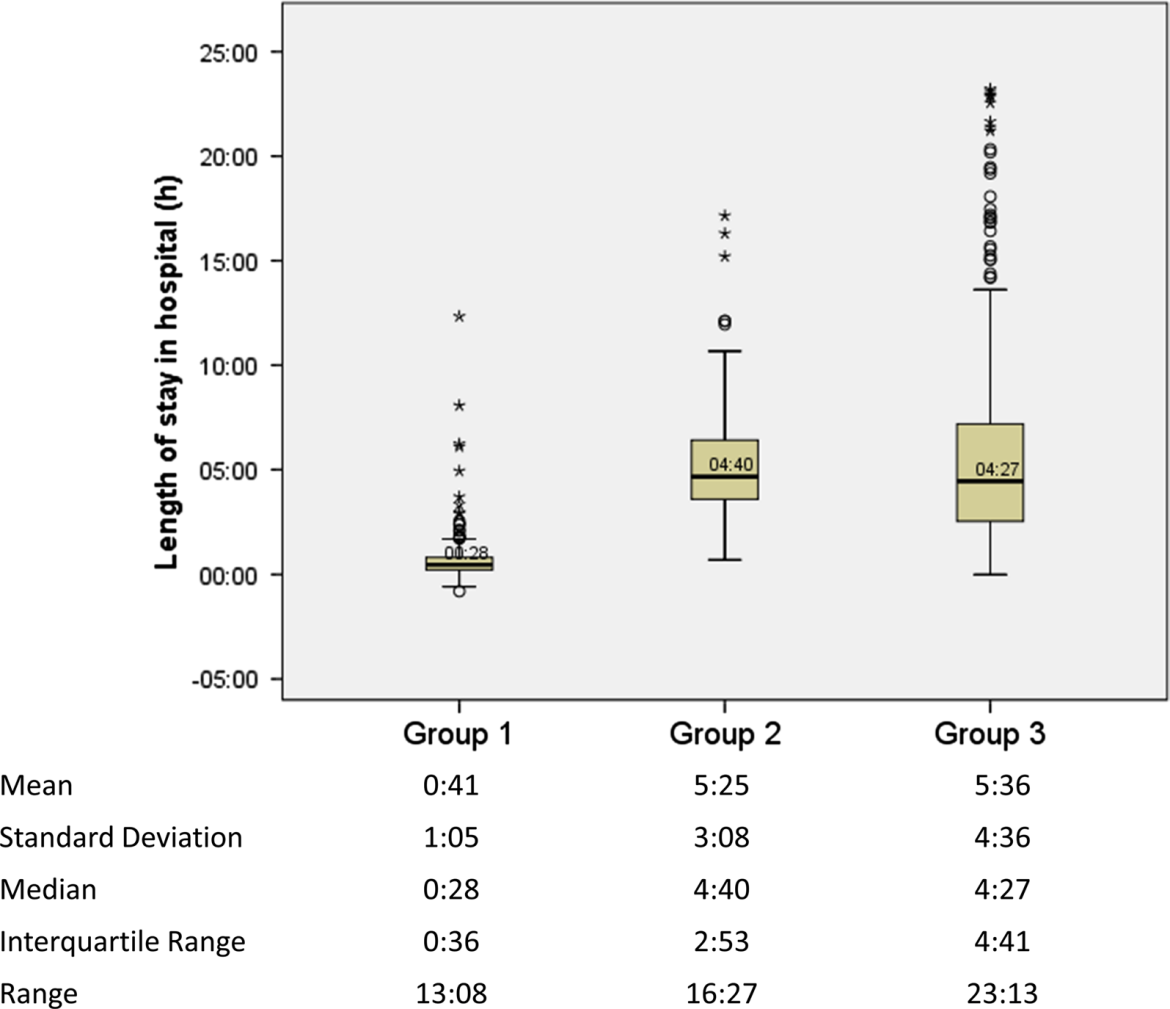
Process length in the 3 clinical groups. Time span was longer and dispersion higher when patients arrived to the emergency department (ED) by themselves (group 3) or when referred to the ED from the primary care high-resolution radiology service (pcHRRS) (group 2) than pcHRRS patients discharged with normal X-rays (group 1). Box upper and lower edges correspond to quartiles 3 (Q3) and 1 (Q1), respectively; ends of the whiskers represent Q3 + 1.5*(Q3 − Q1) and Q1 − 1.5*(Q3 − Q1), respectively; circles and asterisks represent the extreme and very extreme data, respectively (values above or below the whiskers). Group 1 extreme data of 5 h or more correspond to a few specific patients who did not show up at their scheduled time but did so later and even on the following day

**Fig. 4:**
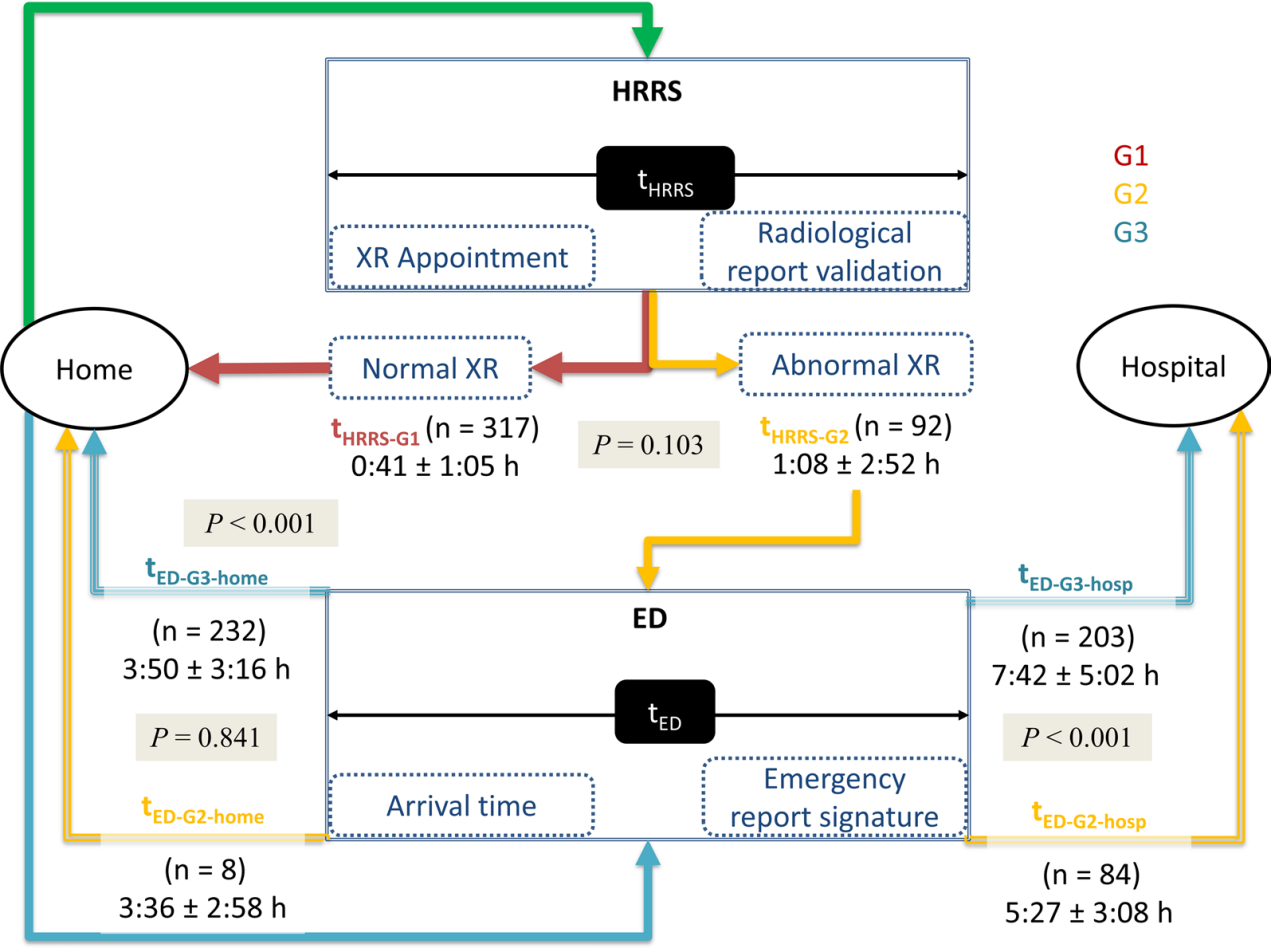
Differences in the process length in each clinical group (G). Decision for G1 patients was always discharge; for G2 and G3 patients could be discharge (home) or hospital admission (hosp). Time length in G1 was always shorter than in G3, regardless of whether G3 patients could return home. Time length in G2 was always shorter than in G3, whatever the decision, even when *t*_pcHRRS_ was added to the *t*_ED_, except for the 9 patients arriving from the pcHRRS who were finally discharged. The process length for most G2 patients was probably shorter since the emergency physicians were notified immediately about the X-ray findings. In those cases, the validation of the radiological report was often delayed, so *t*_pcHRRS-G2_ and *t*_ED-G2_ overlapped in most cases. *t*_pcHRRS_: time span through the primary care high-resolution radiology service (pcHRRS) from the X-ray (XR) appointment to the radiological report validation; *t*_ED_: time span in the emergency department (ED) from the arrival to the clinical report signature

**Fig. 5:**
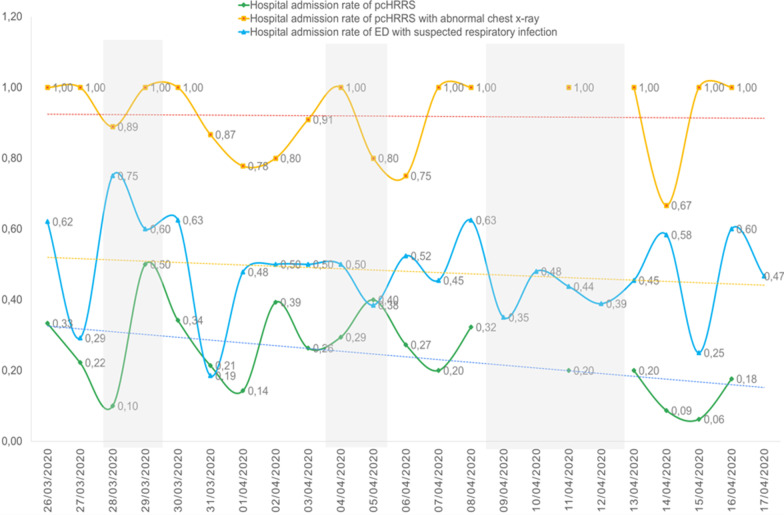
Daily hospital admission rate for the different groups: patients seen through the primary care high-resolution radiology service (pcHRRS, green line), those arriving to the emergency department (ED, blue line) by themselves, and those seen through the pcHRRS but later referred to the ED due to pneumonia findings (yellow line). Grey bands correspond to holidays, when despite being operational, patients were less appointed for a pcHRRS. On March 9th, no patient was appointed for a pcHRRS; on March 10th and 12th, pcHRRS patients did not require admission

After starting the pcHRRS, the number of patients/day in the ED gradually decreased (Fig. 2). G1 patients stayed in hospital significantly less time than G2 and G3 subjects (0:41 ± 1:05 h; 5:25 ± 3:08 h; 5:36 ± 4:36 h, respectively; *p* < 0.001; Fig. 3), even when G2 and G3 patients returned home (0:41 ± 1:05 h; 3:36 ± 2:58 h; 3:50 ± 3:16 h, respectively; *p* < 0.001; Fig. 4). The time spent in the ED did not differ between G2 and G3 when they returned home (3:36 ± 2:58 h vs. 3:50 ± 3:16 h; *p* = 0.841), but was significantly shorter for G2 when patients were admitted (5:27 ± 3:08 h vs. 7:42 ± 5:02 h; *p* < 0.001; Fig. 4). Even considering the pcHRRS and ED times together in G2 patients, they waited less time than G3 patients, though the few 8/92 (8.7%) G2 patients returning home stayed a mean 1.08 + 3.36 h vs. 3:50 h (Fig. 4). The pcHRRS had a high yield for admission decisions, considering that G2 patients were admitted (84/93, 90.3%) more frequently than G3 (203/431, 47.1%; *p* < 0.001), with a rate per day always higher for G2 (mean rates: G2 0.92, range 0.67–1; G3 0.48, range 0.18–0.75), regardless of the epidemics time point (Fig. 5). Moreover, there were no significant differences in the ED radiology times from the electronic X-rays request to the X-ray examination (45.5 ± 57.7 min vs. 51 ± 41.3 min; *p* = 0.396), the X-ray examination to the report validation (46.3 ± 71.1 min vs. 41.8 ± 51.7 min; *p* = 0.569) and the X-rays request to the validation report (91.82 ± 89 min vs. 92.73 ± 67.8 min; *p* = 0.928) during the 5 days before and after the pcHRRS was started.

All 418 pcHRRS patients underwent posteroanterior and lateral chest X-ray views. Oximetry was implemented on March 31st, being applied systematically from patient 109. After getting empirically good results in four patients, 3DDT was systematically performed in addition to chest X-rays since April 1st, starting with patient 152. Regarding the 212 consecutive patients with all data available, their mean age was 46.75 ± 13.93 years, 87 (41%) men. Forty-eight (22.64%), 148 (69.81%) and 16 (7.54%) had abnormal, normal and questionable chest X-ray, respectively. All 54 patients with abnormal radiology examinations were referred to the ED. These 54 patients (G2) were older (50.46 ± 15.73 years) than G1 patients (45.41 ± 13.01 years; *p* = 0.019), men showing a trend to be more frequently referred to the ED than women (29/87, 33.33% vs. 27/115, 23.48%; *p* = 0.057) due to radiological abnormalities.

Among those first consecutive 212 patients, a follow-up chest X-ray was requested in 61 cases. That request was less frequent when initial chest X-ray was normal than when abnormal or questionable (18/148, 12.2% vs. 38/48, 75% vs. 5/16, 31.2%; *p* < 0.001). On the other hand, though follow-up radiographs worsen, respectively, in 15/48 (31.2%) and 2/16 (12.5%) patients with initial abnormal or questionable chest X-ray, no initial normal chest X-ray did it (0/148, 0%) (*p* < 0.001). No G1 patient needed hospital admission during the following weeks. Seven worsened clinically according to their GPs, but neither chest X-rays performed in the following 48 h worsened, nor did those carried out in four of them after the first 48 h. None of those patients required hospital admission. The 29 G2 patients who required hospitalisation for two days or more, and the four patients who required admission to the ICU, already had radiological findings suggestive of pneumonia in the first chest X-ray examination.

Oximetry was performed in 107/212 patients; in 6/212, data were missing. Mean blood oxygen saturation was lower in patients with abnormal (97.24 ± 1.52%, *n* = 17 vs. 98.49 ± 0.88%, *n* = 75; *p* < 0.001) or questionable chest X-ray (98.27 ± 0.80%, *n* = 15; *p* < 0.013).

## Discussion

During the first COVID-19 epidemic wave, respiratory patients were managed significantly faster through the pcHRRS, regardless of whether they returned home or were admitted. The pcHRRS contributed to halve the number of patients arriving to the ED for respiratory symptoms, triaging effectively the need for admission out of the ED.

The screening strategy recommended by the American College of Radiology and the Society of Thoracic Radiology for SARS-CoV-2 infection [6], based on RT-PCR and serology tests, has been applied in some countries [7]. But resources for laboratory tests in suspected SARS-CoV-2 infection were scarce and urged us to manage patients as potential infections in most occasions. Whatever the clinical setting, the RD was strategic when SARS-CoV-2 pneumonia had to be ruled out because hospital admission was then usually warranted [8]. Moreover, providing care for all suspected cases on a hospital basis may likely increase the number of infections in an already overwhelmed health system [7]. Therefore, according to our experience, a radiology entry-door may play a central role in ongoing waves of COVID-19 as our RD provided a faster and more efficient management, which was potentially safer [4].

The RD role to assess need for hospital admission in confirmed or possible COVID-19 patients has been a key innovative efficient strategy, but it was applied without a specific referral from the primary care network, and used CT as imaging technique [9]. Accordingly, the way home-confinement patients were managed and the straightforward imaging assessment are important differences in our case. Potential reduction in infection spread with our pcHRRS might have been achieved by keeping ppCOVID-19 outpatients with respiratory infection isolated in a specific route from the beginning [10, 11]. But, not less important, a personalised agenda contributes to clear facilities [12], guaranteeing a safe environment both for patients and for health workers [9, 11, 13–15]. Risk contacts were significantly reduced through our pcHRRS by providing an extremely quick response in terms of specific appointments, safer X-ray technology and oximetry for decision-making, and expedited information transmission, always out of the ED. At the same time, it safely discriminated patients needing admission from those still ready to be managed at home. More than ¾ of ppCOVID-19 patients managed through the pcHRRS returned home and only 12% of those cases needed further X-rays assessment, who never showed radiological worsening. ppCOVID-19 patients seen through the pcHRRS and the ED routes were discharged in 77% and 52%, respectively, but the pcHRRS route was 5.6 times faster. Moreover, it had a remarkably high performance for discriminating ppCOVID-19 patients needing hospital admission, as over 90% of those referred to the ED were admitted. In a pandemic setting, chest CT has demonstrated high sensitivity but low specificity [16], except for expert radiologists [17]. However, the diagnosis of ppCOVID-19 in our environment has been and continues to be performed by chest X-ray examinations, according to the indications of reference scientific societies [18, 19]. CT scans have been exceptionally used for diagnosis, in severe symptomatic patients with X-ray findings not consistent with the clinical situation or equivocal. Indeed, some authors have agreed on the discriminating potential of chest X-ray as an independent factor for hospital admission [8]. In our case, that performance remained at the same level both at the epidemic peak and while it was declining.

Following our legal obligation [20], we have to keep a safe specific route for patients potentially infected by SARS-CoV-2, which necessarily needs X-ray assessment. We think that our RD workflow for ppCOVID-19 outpatients is this study’s main strength as we haven’t found reported a similar approach. Furthermore, the characteristics of age, gender and oximetry of our G2 patients were similar to those previously reported [8, 21], and the way both cohorts were finally built up within a strict home-confinement regional strategy reproduces a real setting whose results might be used as a model in ongoing pandemic waves. Furthermore, we believe that the higher pcHRRS discriminative performance makes beneficial to maintain and expand the RD entry-door role in the long term to be prepared for future COVID-19 waves or other similar pandemics, even more considering that the pcHRRS didn’t delay our usual radiology workflow of ED patients. But we also acknowledge some weaknesses. We have now complete clinical and radiological follow-up data in 212 patients, so many final outcomes are still under assessment. Although we didn’t consider for decisions any other clinical data beyond chest X-rays and oximetry during patients’ stay at our department, the clinical severity was established by the GPs the same day of the pcHRRS appointment and, when returning home, GPs closely followed them up. Furthermore, blood oxygen saturation levels were normal or almost normal in G1 and G2 groups, probable because our sample was mainly composed by mild forms of the disease. Therefore, we can assume that our decisions were safe. Furthermore, a retrospective assessment of the pcHRRS X-rays examinations by independent staff radiologists is still waiting. But the radiological reports were always validated by board-certified radiologists in patients without pneumonia findings, who never needed admission regardless of whether they were occasionally reassessed. Finally, we couldn’t compare the economic issues at this moment, but, in principle, only a resident without any additional income was required in the pcHRRS, while patients navigating through the ED would normally increase expenses due to other laboratory analysis and medical variability. In this manuscript, we present neither results on clinical–radiological results of our patients, including a Brixia Score analysis, nor data on 3DDT performance. That information was out of the scope of the current manuscript and will be presented in future submissions.

## Conclusion

In summary, a RD positioned as a COVID-19 entry-door may be efficient in an epidemic setting to decrease respiratory patients at the ED, while potentially reducing infection hazards through safe and expedited decisions.

## Supplementary Information

**Additional file 1: Supplementary figure 1.** Primary Care High-resolution radiology service (pcHRRS) organisation. Patients confined at home with possible or confirmed SARS-CoV-2 infection, monitored by telephone by their General Practitioners (A) needing radiology assessment were appointed within the next 24h (B) and supported by the radiology secretary staff (C). X-rays and blood oxygen saturation (BOS) were obtained at the Radiology Department (D) and an immediate radiological report emitted, including BOS and the patient’s final destination according to the radiological findings. Destination could be the Emergency Department or home confinement depending on radiological signs of pneumonia (E). **Supplementary figure 2.** Instructions for the primary care high-resolution radiology service patient by the administrative staff. **Supplementary figure 3.** Decision-making algorithm performed by the radiology resident and supervised by the staff radiologist. Abnormal chest x-ray: radiological findings suggestive of pneumonia: ground glass opacities or consolidations with or without reticular pattern in a patient with symptoms of respiratory infection; normal chest x-ray: absence of the radiological findings suggestive of pneumonia; questionable chest x-ray: uncertain radiological findings. BOS: blood oxygen saturation. **Supplementary figure 4.** Structured Radiological Report. **Supplementary figure 5.** Flow chart of the included and excluded patients.

## Data Availability

The datasets used and/or analysed during the current study are available from the corresponding author on reasonable request.

## Abbreviations

3DDT: Digital 3D tomosynthesis
ED: Emergency department
G1: Group 1
G2: Group 2
G3: Group 3
GP: General practitioner
pcHRRS: Primary care high-resolution radiology service
ppCOVID-19: Possible pneumonia COVID-19
RD: Radiology department
