# Supplementary material for: Radiology departments as COVID-19 entry-door might improve healthcare efficacy and efficiency, and emergency department safety
Source: Insights Imaging. 2021 Jan 4;12:1. doi: 10.1186/s13244-020-00954-8 (PMC7781166; doi:10.1186/s13244-020-00954-8)
Supplement: Supplementary file 1 — Additional file 1: Supplementary figure 1. Primary Care High-resolution radiology service (pcHRRS) organisation. Patients confined at home with possible or confirmed SARS-CoV-2 infection, monitored by telephone by their General Practitioners (A) needing radiology assessment were appointed within the next 24h (B) and supported by the radiology secretary staff (C). X-rays and blood oxygen saturation (BOS) were obtained at the Radiology Department (D) and an immediate radiological report emitted, including BOS and the patient’s final destination according to the radiological findings. Destination could be the Emergency Department or home confinement depending on radiological signs of pneumonia (E). Supplementary figure 2. Instructions for the primary care high-resolution radiology service patient by the administrative staff. Supplementary figure 3. Decision-making algorithm performed by the radiology resident and supervised by the staff radiologist. Abnormal chest x-ray: radiological findings suggestive of pneumonia: ground glass opacities or consolidations with or without reticular pattern in a patient with symptoms of respiratory infection; normal chest x-ray: absence of the radiological findings suggestive of pneumonia; questionable chest x-ray: uncertain radiological findings. BOS: blood oxygen saturation. Supplementary figure 4. Structured Radiological Report. Supplementary figure 5. Flow chart of the included and excluded patients. [file 13244_2020_954_MOESM1_ESM.docx]

**ELECTRONIC SUPPLEMENTARY MATERIAL**

**Supplementary figures**

**
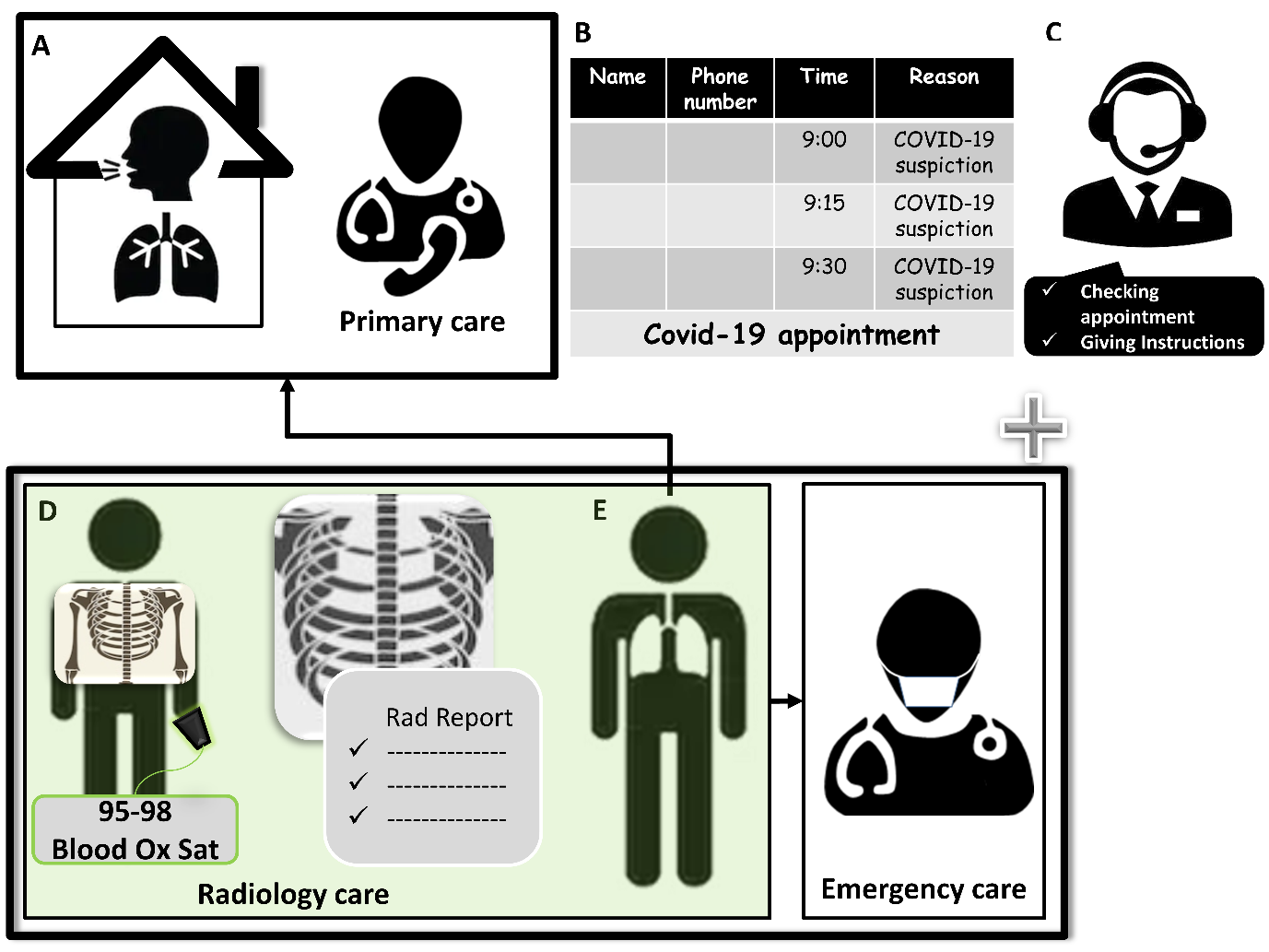
**

**Supplementary figure 1**. Primary Care High-resolution radiology service (pcHRRS) organization. Patients confined at home with possible or confirmed SARS-CoV-2 infection, monitored by telephone by their General Practitioners (A) needing radiology assessment were appointed within the next 24h (B) and supported by the radiology secretary staff (C). X-rays and blood oxygen saturation (BOS) were obtained at the Radiology Department (D) and an immediate radiological report emitted, including BOS and the patient’s final destination according to the radiological findings. Destination could be the Emergency Department or home confinement depending on radiological signs of pneumonia (E).


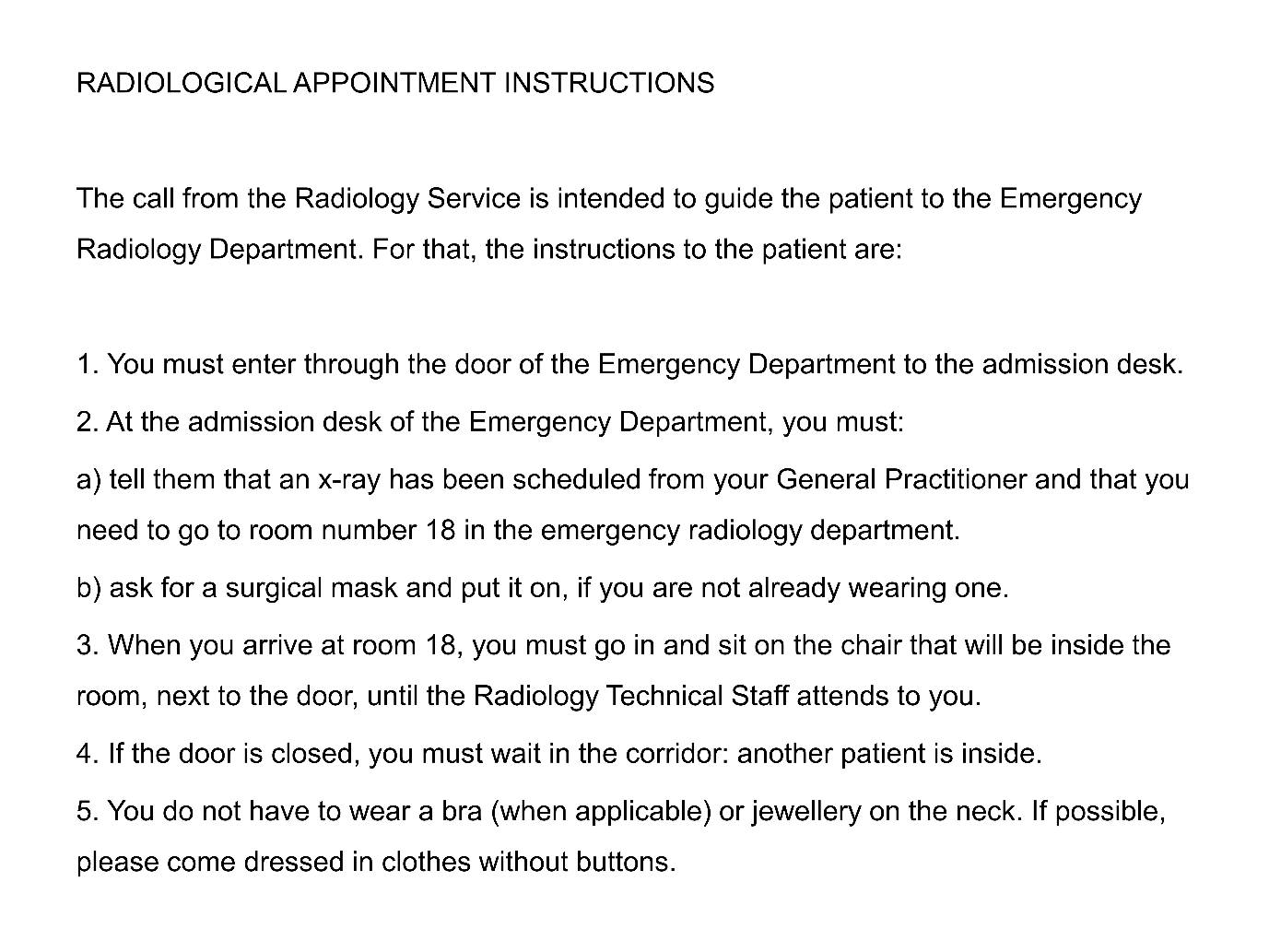


**Supplementary figure 2.** Instructions for the primary care high-resolution radiology service patient by the administrative staff.


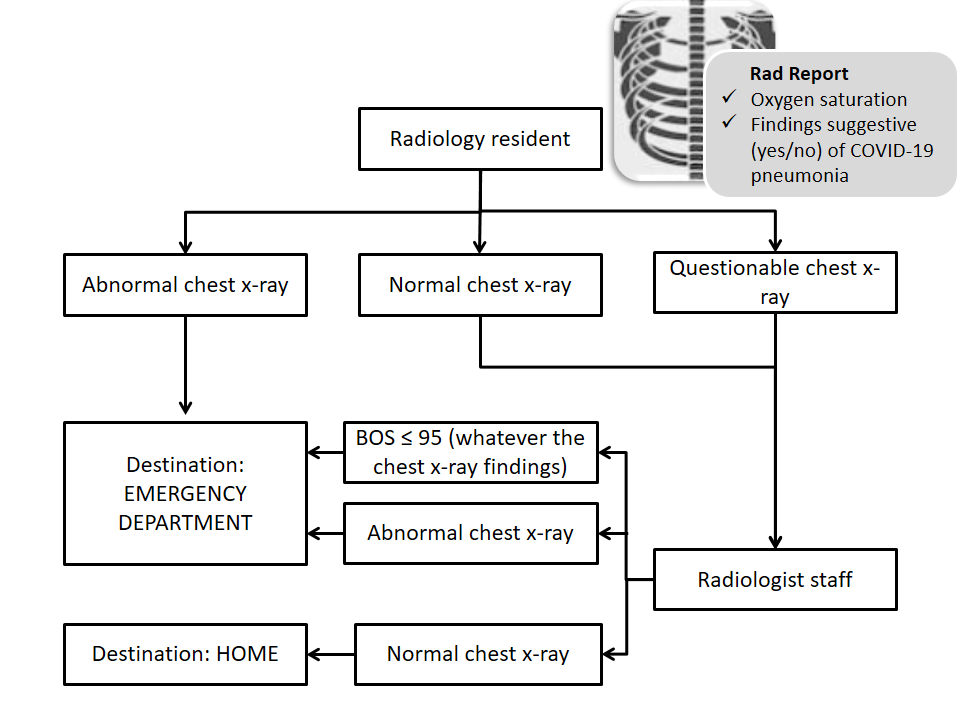


**Supplementary figure 3**. Decision-making algorithm performed by the radiology resident and supervised by the staff radiologist.

Abnormal chest x-ray: radiological findings suggestive of pneumonia: ground glass opacities or consolidations with or without reticular pattern in a patient with symptoms of respiratory infection; normal chest x-ray: absence of the radiological findings suggestive of pneumonia; questionable chest x-ray: uncertain radiological findings.

BOS: blood oxygen saturation.


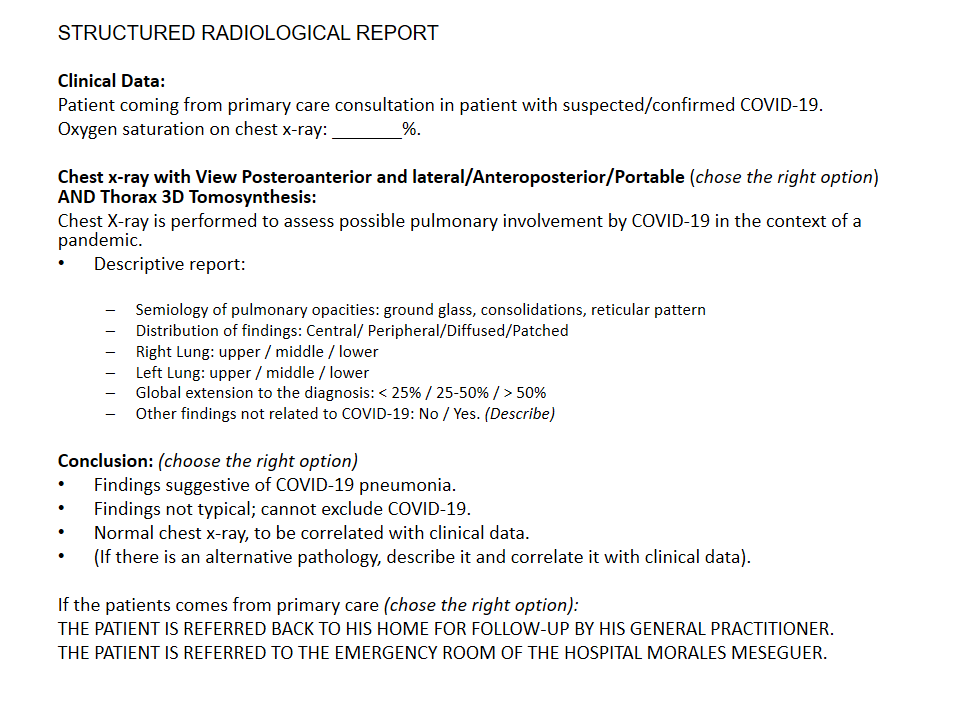


**Supplementary figure 4**. Structured Radiological Report.


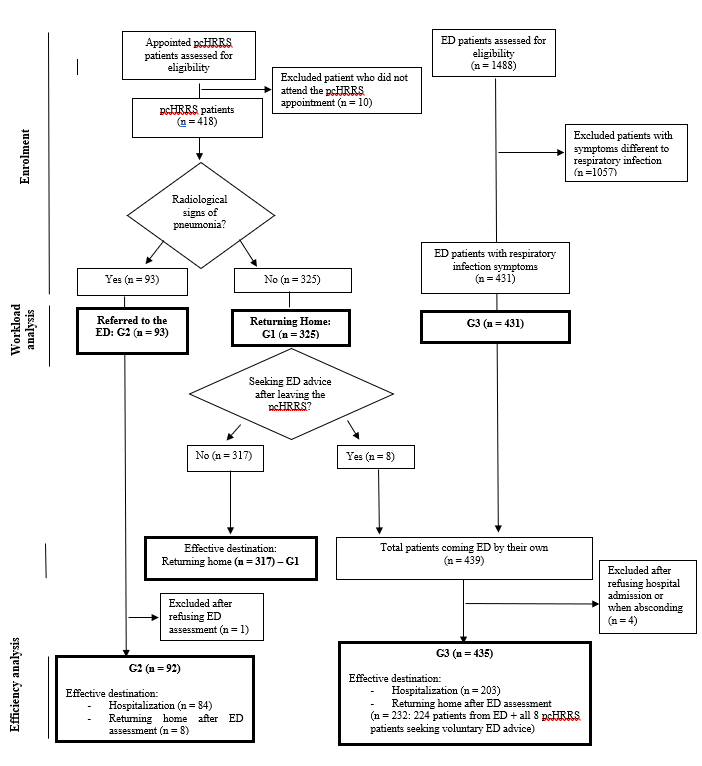


**Supplementary figure 5.** Flow chart of the included and excluded patients.
